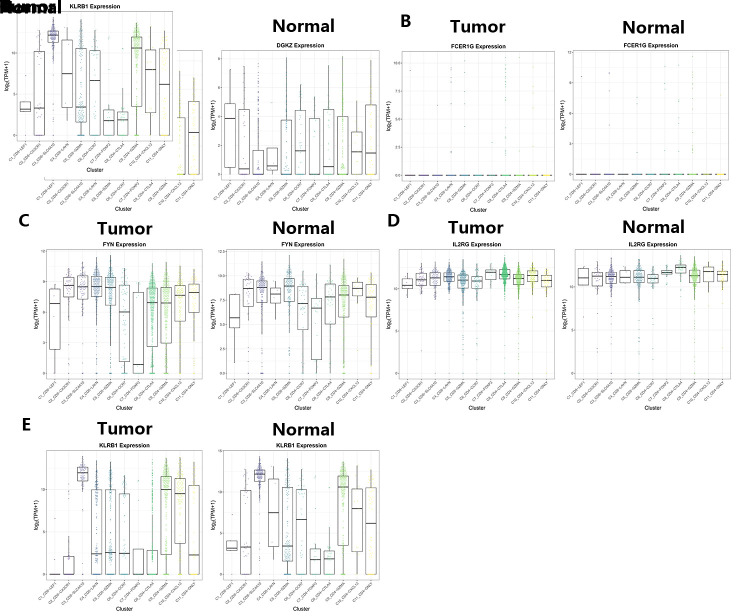# A novel CD8+ T cell-related gene signature as a prognostic biomarker in hepatocellular carcinoma

**DOI:** 10.1097/MD.0000000000037496

**Published:** 2024-03-15

**Authors:** Xiaozhen Peng, Xingjun Lu, Daqing Yang, Jinyan Liu, Honglin Wu, Hong Peng, Yiya Zhang

**Affiliations:** a School of Public Health & Laboratory Medicine, Hunan University of Medicine, Huaihua, China; b Hunan Provincial Key Laboratory for Synthetic Biology of Traditional Chinese Medicine, Hunan University of Medicine, Huaihua, China; c Hunan Normal University, Changsha, China; d Medical School, Huanghe Science & Technology College, Zhengzhou, China; e Department of Dermatology, Xiangya Hospital, Central South University, Changsha, China.

**Keywords:** CD8+ T cell, hepatocellular carcinoma, immune-related, infiltration-related gene, therapeutic response

## Abstract

CD8+ T cells have great roles in tumor suppression and elimination of various tumors including hepatocellular carcinoma (HCC). Nonetheless, potential prognostic roles of CD8+ T cell-related genes (CD8Gs) in HCC remains unknown. In our study, 416 CD8Gs were identified in HCC, which were enriched in inflammatory and immune signaling pathways. Using The Cancer Genome Atlas dataset, a 5-CD8Gs risk model (KLRB1, FYN, IL2RG, FCER1G, and DGKZ) was constructed, which was verified in International Cancer Genome Consortium and gene expression omnibus datasets. Furthermore, we found that overall survival was independently correlated with the CD8Gs signature, and it was associated with immune- and cancer-related signaling pathways and immune cells infiltration. Finally, drug sensitivity data indicated that 10 chemotherapeutic drugs held promise as therapeutics for HCC patients with high-risk. In conclusion, multi-databases analysis showed that 5-CD8Gs and their signature could be an indicator to predict candidate drugs for HCC therapy.

## 1. Introduction

Hepatocellular carcinoma (HCC) as the major common primary malignancies remains the leading cause of cancer-related death.^[1]^ Despite significant advances in diagnosis and therapy, HCC represents approximately 90% of primary liver cancers. Surgery is no longer applicable at a late-stage because of the high rate of intra- or extrahepatic metastases and recurrence.^[2–4]^ Extensive efforts have been made to develop immunotherapy as a novel treatment for patients with HCC. Thus, screening for novel immune hallmarks and optimizing precision immunotherapy of HCC patients are essential for improving prognosis.

CD8-expressing (CD8+) lymphocytes are crucial roles in suppressing tumor growth and mediate tumors elimination.^[5–7]^ CD8+ tumor-infiltrating lymphocytes (TILs) have been extensively described and identified as prognostic markers in a number of solid tumors.^[8–13]^ Based on the high power of the natural immune system, immunotherapy is a powerful strategy for tumor treatment, including HCC.^[14–16]^ The available immunotherapies are applicable in limited situations in which CD8+ T cell infiltration is highly expressed in HCC,^[17–20]^ and strategies for enhancing CD8+ T cell priming and accumulation perform improved antitumor efficacy.^[20,21]^ However, no studies have sought to systematically describe the potential roles of CD8+ T cell-related genes (CD8Gs) in predicting HCC prognosis and the immunotherapies.

In this study, we obtained 26 prognosis-related CD8Gs. Next, 5-CD8Gs risk model was constructed and it was correlated with a better prognosis for HCC. The CD8Gs signature was associated with the immune cell infiltration and 10 drugs were identified as sensitive drugs for HCC with high-risk. In a word, this work illustrated the CD8Gs signature for predicting the prognosis and drug sensitivity of HCC patients.

## 2. Materials and methods

We obtained gene expression data as well as clinical information from The Cancer Genome Atlas (TCGA) (https://protal.gdc.cancer.gov/) and International Cancer Genome Consortium (ICGC) datasets for HCC patients.

Somatic mutation profiles were downloaded from the TCGA data portal (https://portal.gdc.cancer.gov/). The somatic mutation data was processed with the R package “maftools.”

### 2.1. Construction of a prognostic model

In this study, calculations on immune cells in HCC were performed using XCELL and CIBERSORT. Then, we identified stable expressed genes with *R* > 0.4 and *P* < .05 using Pearson correlation analysis, which were considered as CD8+ T cell-related genes. Furthermore, prognosis-related CD8Gs were identified using LASSO regression and Cox analysis in the TCGA database. Then, the ICGC dataset was used to verify the CD8G signatures. Finally, we constructed a risk score as follows: coefficient1 × gene-1 + coefficient2 × gene-2 + coefficient3 × gene-3 + ….

The predicting survival outcomes of the low-risk and high-risk groups were assessed using R package “survival” and log-rank test in Kaplan–Meier survival curves.

To explore the accuracy of the predictive performance, we applied the survival receiver operating characteristic (ROC) curve. To calculate the prognostic value of the CD8Gs signature, we used the R package “survival.”

We constructed a nomogram from each cohort’s risk score, and other clinical parameters. ROC curves were generated to compare clinical characteristics with risk scores via the R software “ROC package.”

### 2.2. Function analysis and enrichment analysis

Gene set enrichment analysis was performed to reveal the functional enrichment of CD8Gs. Gene Ontology analyses and Kyoto Encyclopedia of Genes and Genomes enrichment were utilized to explore pathways related to CD8Gs.

### 2.3. The risk-related CD8Gs expression in the immune cells of HCC

We performed the tSNE analysis using web tools (http://hcc.cancer-pku.cn/) to identify the expression of risk-related CD8Gs in immune cells.

### 2.4. Drug sensitivity

To predict the risk of immunotherapy for patients with HCC, we calculated IC_50_ of administered chemotherapeutic drugs in the TCGA project of the LIHC dataset. Besides, we evaluated IC_50_ of drugs between low- and high-risk groups using Wilcoxon signed-rank test, and Prophetic and ggplot2 of R were used to obtain box drawings.

### 2.5. Statistical analysis

Survival analysis was performed using the Kaplan–Meier method. LASSO and Cox regression were used to analyze the relationship between the CD8Gs signature and the clinical parameters of patients with HCC. Statistical analyses were conducted in the R language.

## 3. Results

### 3.1. CD8+ T cells infiltration in HCC

**Figure 1.**
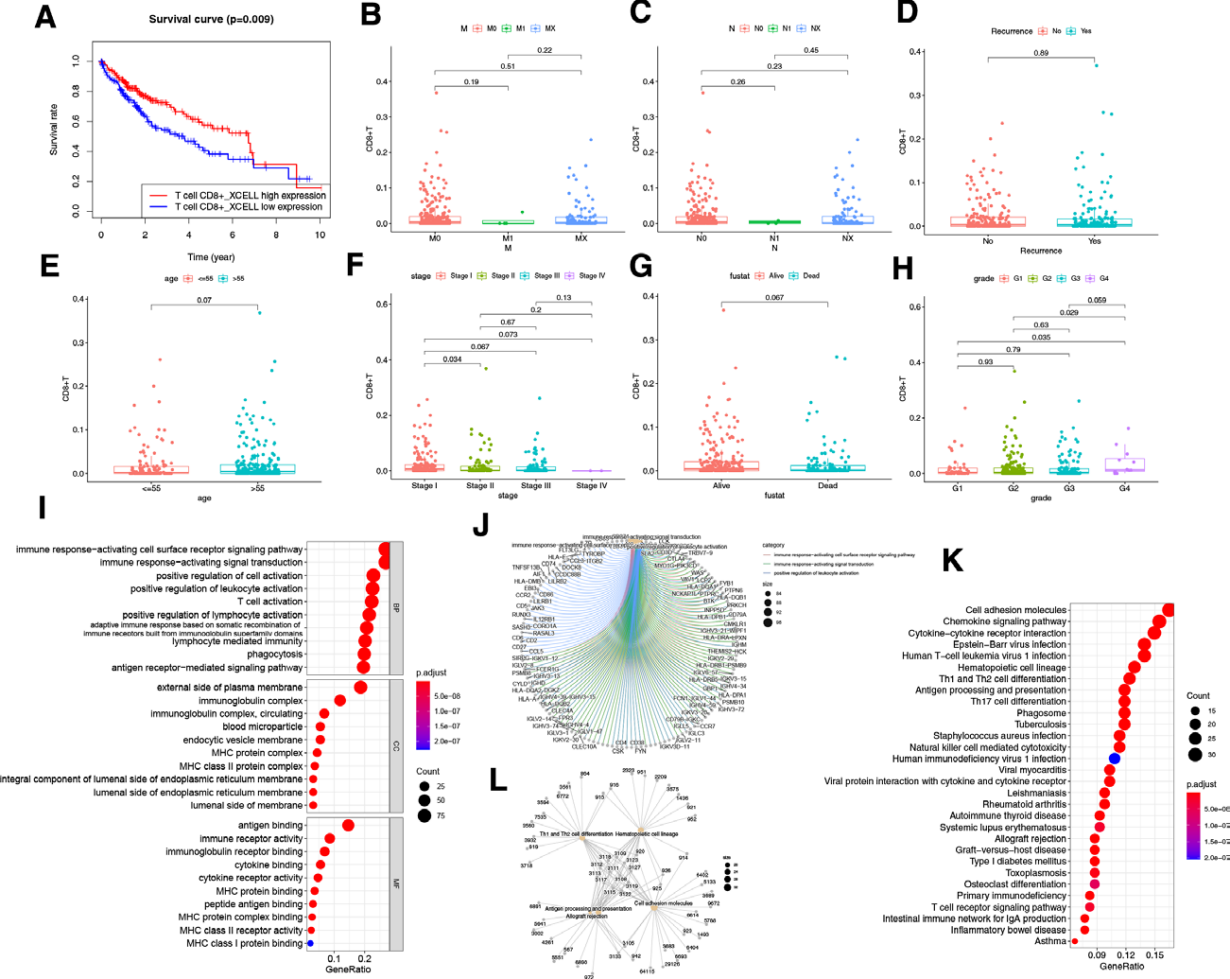
Cells infiltration. Relationship between CD8+ T cell expression and survival rate (A), and clinical characteristics (B–H). (I–K) The enrichment pathways of CD8Gs. GO analysis (I and J) and KEGG analysis (K and L). GO = gene ontology, KEGG = Kyoto encyclopedia of genes and genomes.

Patients were divided into low- and high-expression groups based on the median of CD8+ infiltration. Figure 1A showed that patients with low infiltration of CD8+ T cells had a lower survival rate (Kaplan–Meier analysis). Figure 1B–H described a correlation between CD8+ T cell infiltration and clinical characteristics. The results indicated that CD8+ T cell infiltration was prominently decreased in stage II compared to that in stage I (Fig. 1F). CD8+ T cell infiltration was significantly decreased in G1 compared to G4 and G2 compared to G4 (Fig. 1H). The results indicated that CD8+ T cell infiltration was correlated with stage and grade. However, CD8+ T cells number was not correlated with M, N, recurrence, or age in HCC (Fig. 1B–E and G).

Next, a total of 416 CD8Gs were identified, and Gene Ontology analysis revealed that CD8Gs were mainly enriched in immune response-activating signal transduction, immune response-activating cell surface receptor signaling pathway, and positive regulation of cell activation vital biological processes (Fig. 1I and J). For Kyoto Encyclopedia of Genes and Genomes analysis, CD8Gs were enriched in cell adhesion molecules, chemokine signaling pathways, and cytokine-cytokine receptor interactions (Fig. 1K and L).

### 3.2. Construction and validation of a CD8Gs signature in HCC

**Figure 2.**
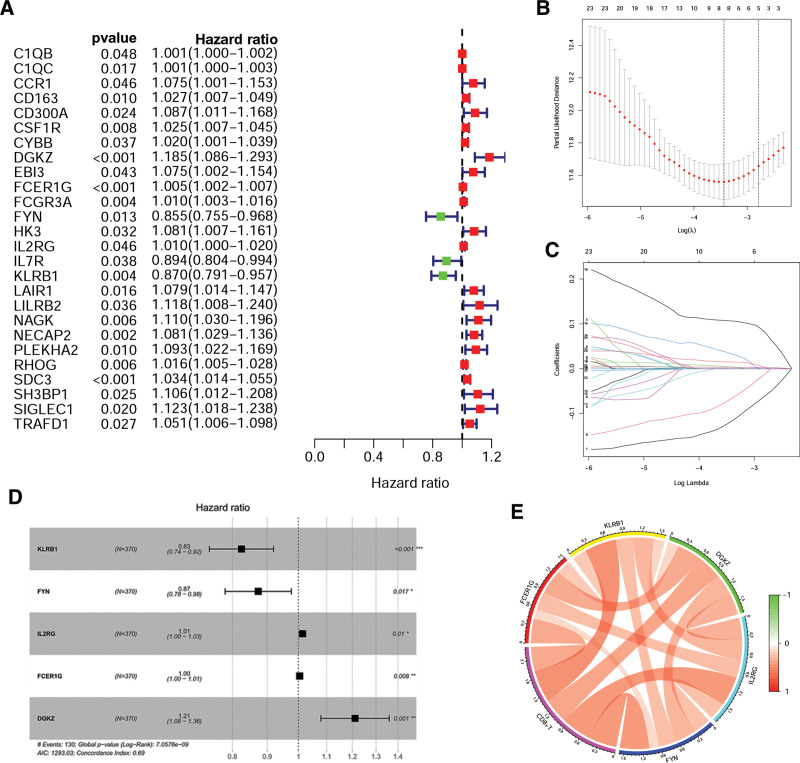
The CD8Gs prognostic signature in HCC. (A) Twenty-six CD8Gs. (B) LASSO coefficient values of CD8Gs in HCC. (C) Profiles of LASSO coefficients. (D) Multivariate cox analysis of CD8Gs. (E) The correlation between the expression of CD8+ T, KLRB1, FYN, IL2RG, FCER1G, and DGKZ. *P < .05, **P < .01, ***P < .001. CD8Gs = CD8+ T cell-related genes, HCC = hepatocellular carcinoma.

Firstly, we used univariate Cox regression analysis to identify 26 prognosis-related CD8Gs (Fig. 2A). Secondly, LASSO regression (Fig. 2B and C) and multivariate Cox analysis (Fig. 2D) were used to construct a risk model based on KLRB1, FYN, IL2RG, FCER1G, and DGKZ. Based on the following formula, the risk score is KLRB1 × −0.1923 + FYN × −0.1357 + IL2RG × 0.0141 + FCER1G × 0.0042 + DGKZ × 0.1916. The survival rate of the CD8Gs expression were showed in Figure S1, Supplemental Digital Content, http://links.lww.com/MD/L894. Figure 2E shows the positive relationship between CD8+ T infiltration and the expression of genes (KLRB1, FYN, IL2RG, FCER1G, and DGKZ).

**Figure 3.**
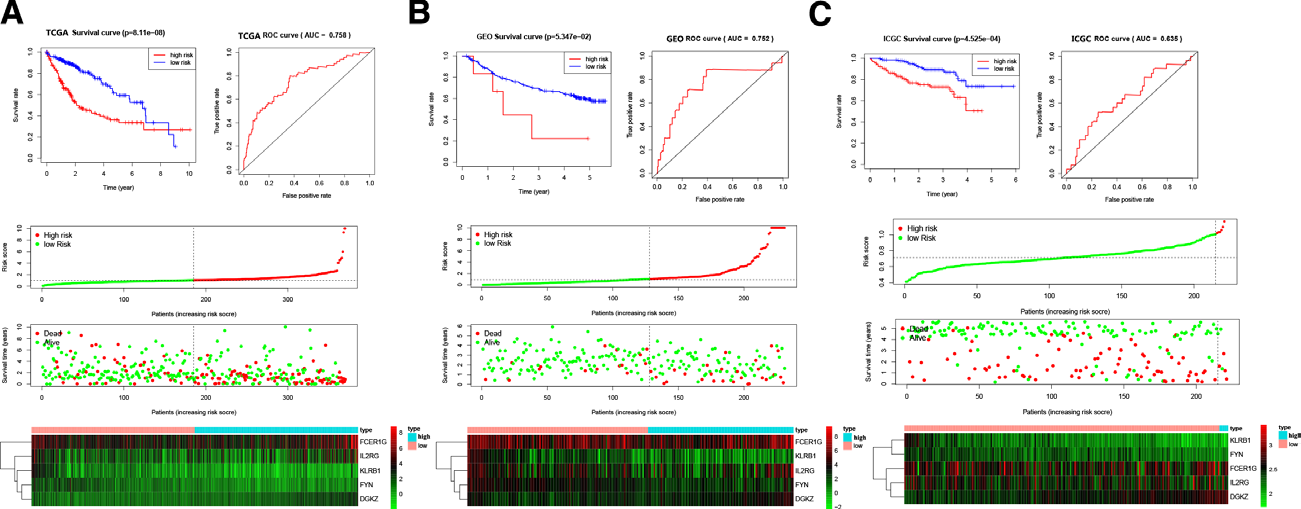
CD8Gs-based risk score in 3 cohorts. Survival analysis, heat map, and risk score of low- and high-risk subgroups were analyzed by the TCGA (A), ICGC (B), and GEO (C) database. CD8Gs = CD8+ T cell-related genes, GEO = gene expression omnibus, ICGC = International Cancer Genome Consortium, TCGA = The Cancer Genome Atlas.

Next, we performed joint analysis of multiple databases to explore the prognostic role of the CD8Gs signature. According to survival analysis, a poor prognosis was observed for patients in the high-risk subgroup, with area under the curve (AUC) values of 0.758 (TCGA dataset), 0.752 Gene Expression Omnibus (GEO dataset), and 0.635 (ICGC dataset), respectively (Fig. 3A–C).

### 3.3. CD8Gs signature served as an independent prognostic marker

**Figure 4.**
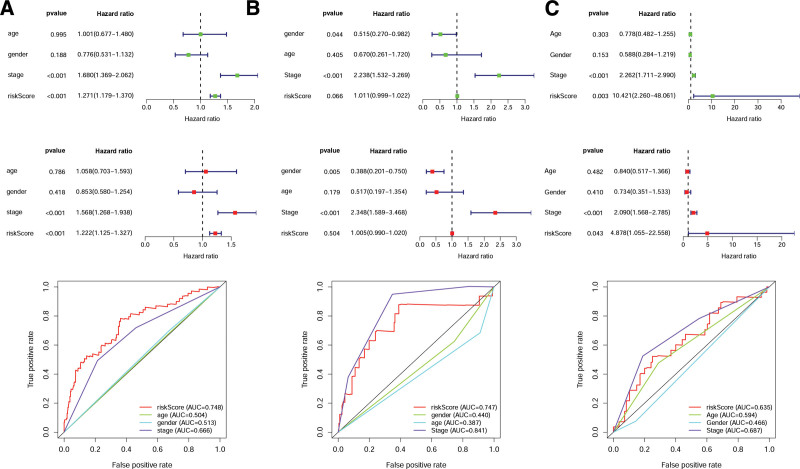
Clinicopathological characteristics of patients with HCC were associated with risk score. The forest plots for univariate, multivariate cox regression analysis, and the areas under the ROC curve about stage, age, risk score, and gender using TCGA databases (A), ICGC databases (B), and GEO databases (C). GEO = gene expression omnibus, HCC = hepatocellular carcinoma, ICGC = International Cancer Genome Consortium, TCGA = The Cancer Genome Atlas.

We next analyzed the prognosis role of CD8Gs signature and clinical characteristics in HCC. Based on univariate cox analysis, the CD8Gs signature was associated with HCC prognosis in TCGA and GEO database. There were no differences in the prognostic factors for the stage based on univariate and multivariate Cox regression analyses, with AUC values of 0.666, 0.841, and 0.687, respectively (Fig. 4). A risk score was proven to be an independent prognostic factor using TCGA and GEO databases, with AUC values of 0.748 and 0.635, respectively (Fig. 4A and C).

**Figure 5.**
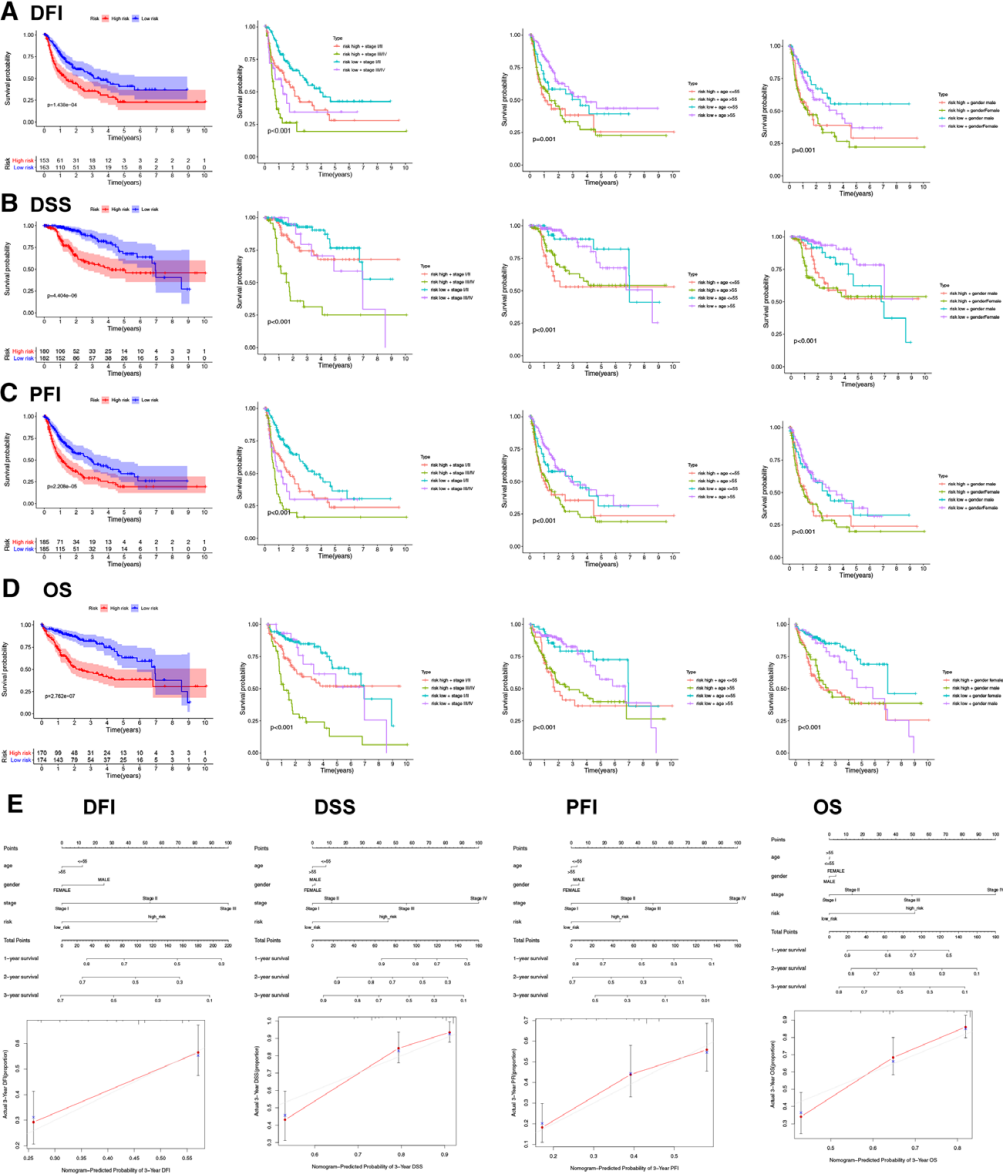
The prognosis of HCC patients with high-/low-risk scores. The correlation between clinicopathological features and DFI (A), DSS (B), PFI (C), and OS (D). The nomogram of 1-, 2-, or 3-year DFI, DSS, PFI, and OS is based on risk score, age, and gender. The predicted and the actual DFI, DSS, PFI, and OS for the prognosis model were evaluated by calibration plots (E). DFI = disease-free interval, DSS = disease-specific survival, HCC = hepatocellular carcinoma, OS = overall survival, PFI = progression-free interval.

To further predict the prognostic ability, disease-specific survival (DSS), disease-free interval (DFI), progression-free interval (PFI), and overall survival (OS) were used to distinguish high- and low-risk HCC patients. We found that DFI, DSS, PFI, and OS were different between high- and low-risk groups, which implied that the CD8Gs model level predicted the prognosis of HCC patients (Fig. 5A–D). According to the subgroups by stage, age, and sex, the DFI, DSS, PFI, and OS of the high-risk subgroup were inferior to that of the low-risk subgroup in the TCGA dataset (Fig. 5A–D). The results indicated that high-risk patients also had significantly shorter DFI, DSS, PFI, and OS in stage III/IV, age > 55, age ≤55, male and female, respectively than patients with low-risk.

We developed a nomogram that predicted patients’ outcomes by incorporating signatures with age, gender, stage, and risk in the TCGA dataset (Fig. 5E). We plotted the calibration curve to evaluate the agreement between the predicted probability of the prognostic model and the actual outcomes. Figure 5E shows the high reliability of the nomogram in the DFI, PFI, DSS, and OS survival of patients with HCC. According to the results, the prognostic model was found to predict survival from HCC.

### 3.4. Genomic alterations

**Figure 6.**
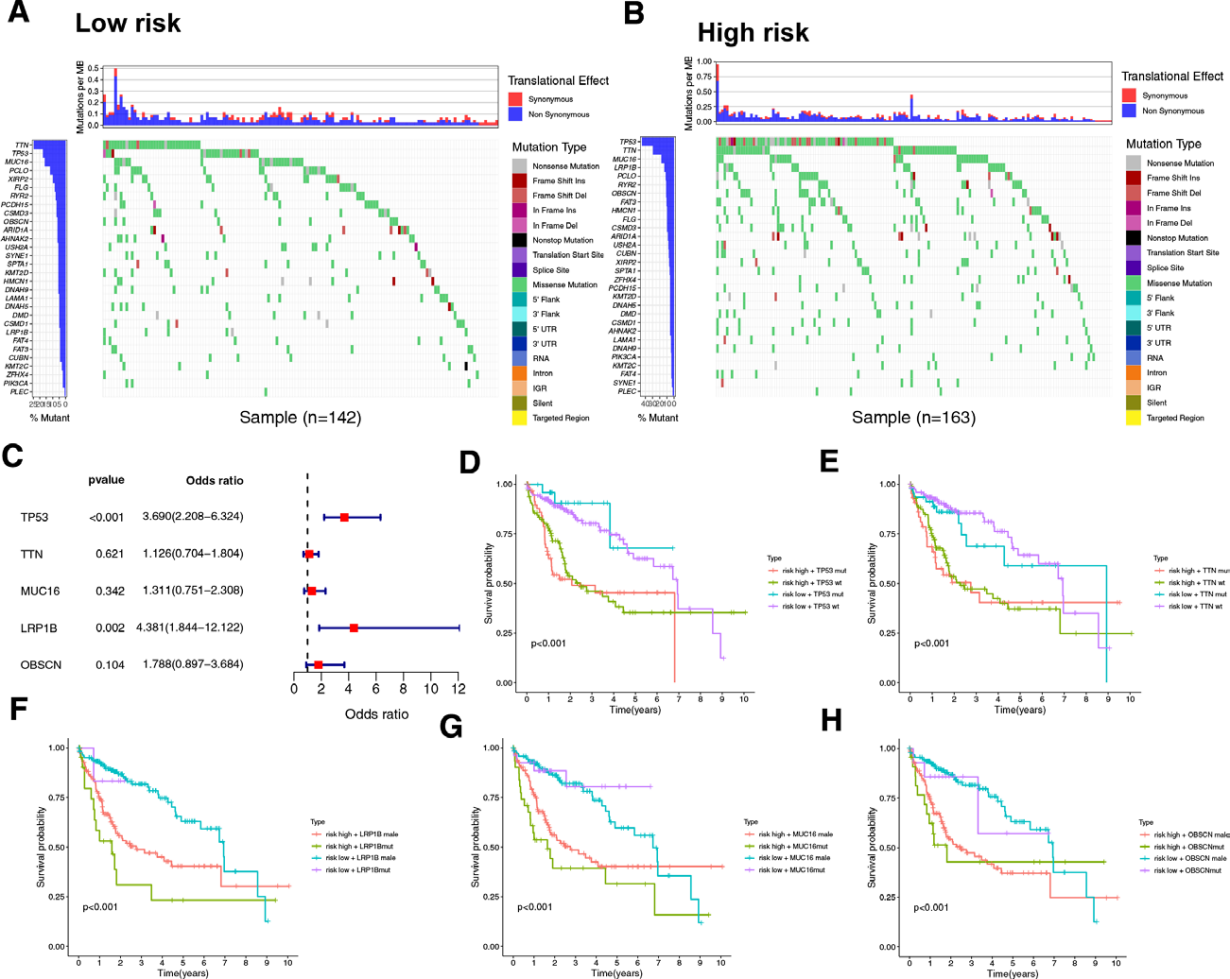
Genomic alterations between different groups. The most frequently mutated (30 top) genes in low- (A) and high-risk (B) groups. (C) Differently mutated genes between 2 cohorts. Independent correlation between overall survival and TP53 (D), TTN (E), LRP1B (F), MUC16 (G), and OBSCN (H) mutation in each cohort.

We next analyzed the relationship between CD8Gs signature and mutations state in HCC. The most frequently mutated (top 30) genes in 2 cohorts were performed in Figure 6A and B. Among these, TP53 and LRP1B mutations were associated with HCC prognosis (Fig. 6C). Furthermore, We analyzed the correlations between mutations in driver genes (TP53, TTN, LRP1B, MUC16, and OBSCN) and survival probability (Fig. 6D–H).

### 3.5. CD8Gs signature was associated with immune infiltration in HCC

**Figure 7.**
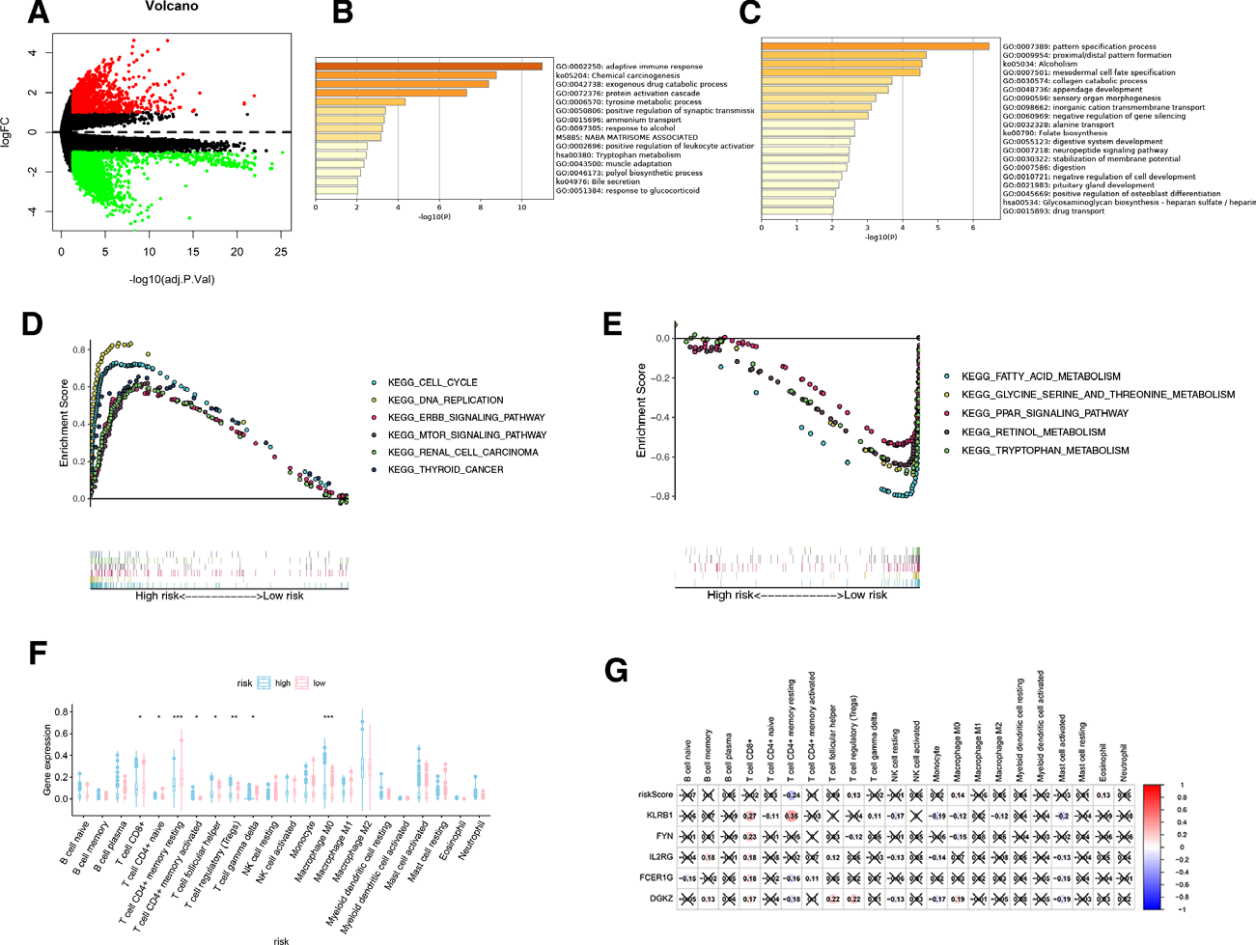
DEGs between low-risk and high-risk groups. (A) Volcano plot of differently mutated genes. GO analysis was conducted for upregulated (B) and downregulated (C) genes. GSEA analysis of the association between immune infiltration and CD8Gs in HCC patients with high- (D) and low-risk (E) scores. (F) Immune cells in the high/low group. (G) The relationship between immune cells and the risk score. *P < .05, **P < .01, ***P < .001. DEGs = differentially expressed genes, GO = gene ontology, GSEA = gene set enrichment analysis, HCC = hepatocellular carcinoma.

To reveal the risk-related signal pathway, 5506 differentially expressed genes, including 4408 downregulated genes and 1152 upregulated genes, were identified in 2 groups with |logFC| > 1 and *P* < .05, respectively (Fig. 7A). Then, we found that upregulated genes were primarily concentrated in pathways including adaptive immune response, chemical carcinogenesis, exogenous drug catabolic process, and protein activation cascade (Fig. 7B), while downregulated genes were primarily concentrated in different metabolic processes (Fig. 7C) using enrichment analysis. Moreover, gene set enrichment analysis showed that the high-risk group was highly enriched in DNA replication, cell cycle, and cancer-related signal pathways (Fig. 7D), while the low-risk was highly correlated with fatty acid metabolism (Fig. 7E). To further reveal the association between risk signature and immune status, CIBERSORT was used to predict the immune cell infiltration in HCC. As shown in Figure 7F, the infiltration of T cell gamma delta, T cell regulatory (Tregs), T cell follicular helper, T cell CD4+ memory activated, T cell CD8+, and Macrophage M0 were increased in HCC patients with high-risk, while T cell CD4+ naive and CD4+ memory resting cells were higher in the low-risk group. Pearson analysis showed that KLRB1 was positively associated with T cell CD4+ memory resting and T cell CD8+, and negatively correlated with mast cell activated; FYN was positively associated with T cell CD8+, and DGKZ was positively associated with T cell regulatory and T cell follicular helper (Fig. 7G).

**Figure 8.**
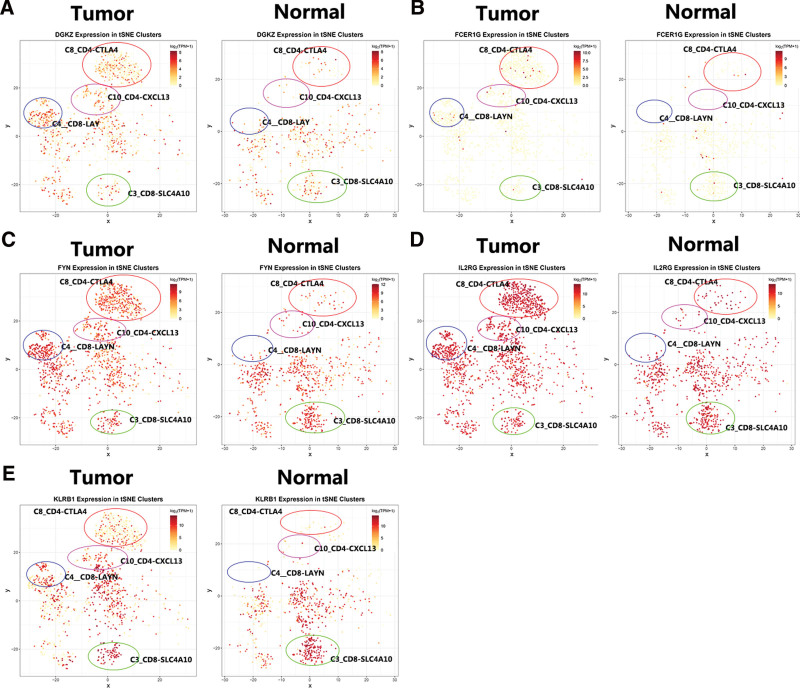
The expression of CD8Gs in HCC. tSNE cluster web tool was used to analyze the expression of DGKZ (A), FCER1G (B), FYN (C), IL2RG (D), and KLRB1 (E) in immune cells. CD8Gs = CD8+ T cell-related genes, HCC = hepatocellular carcinoma.

Then, the single-cell sequencing results were used to reveal the FYN, IL2RG, FCER1G, DGKZ, and KLRB1 expression in immune cells by the tSNE cluster (http://hcc.cancer-pku.cn/) (Fig. 8) and box plots web tool (Figure S2, Supplemental Digital Content, http://links.lww.com/MD/L895). A total of these 5 genes were expressed at greater in the C10_CD4-CXCL13, C4_CD8-LAYN, and C8_CD4-CTLA4 bundle of abnormal tissues than normal tissues, and in the C3_CD8-SLC4A10 bundle of normal liver tissues compared to HCC tissues (Fig. 8).

### 3.6. Analysis of risk model and drug sensitivity

**Figure 9.**
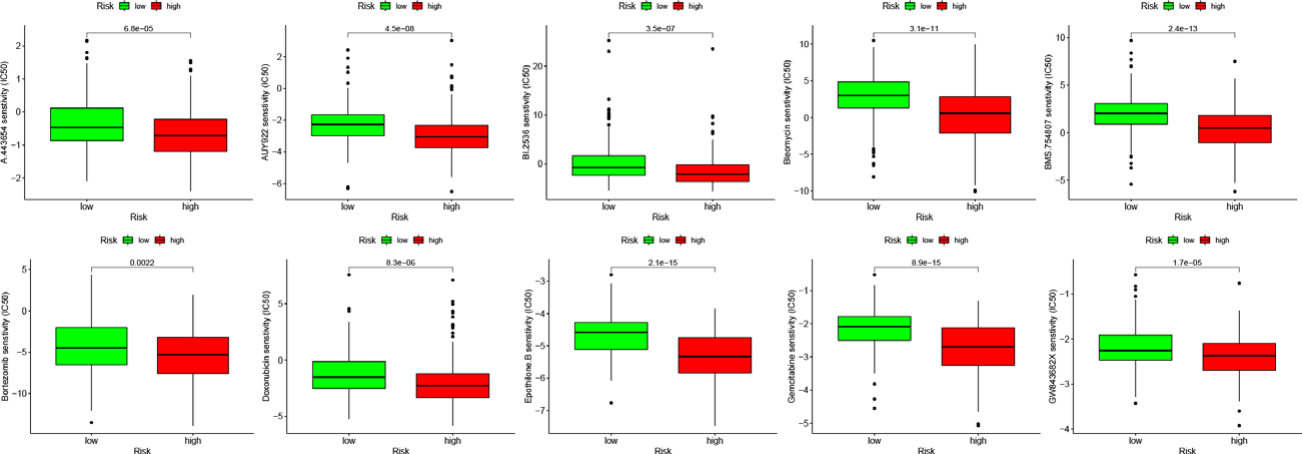
Drug sensitivity of patients with high- and low-risk.

To identify any of the available common drugs used in HCC patient therapy, we analyzed the correlation risk score with drug sensitivity. We found that the high-risk group was more sensitive to drugs, including A.443654 (*P* = 6.8e−05), AUY922 (*P* = 4.5e−08), BI.2536 (*P* = 3.5e−07), bleomycin (*P* = 3.1e−11), BMS.754807 (*P* = 2.4e−13), bortezomib (*P* = .0022), doxorubicin (*P* = 8.3e−06), epothilone B (*P* = 2.1e−15), gemcitabine (*P* = 8.9e−15), and GW843682X (*P* = 1.7e−05) (Fig. 9). It was suggested that the model could be a predictor of the response to drug sensitivity.

## 4. Discussion

CD8+ T cells are essential for tumor elimination and suppression,^[22]^ reflecting the implementation of adaptive immune responses and tumor-specific immune responses against cancer, rendering them suitable targets for immunotherapy.^[23,24]^ The success of adoptive cellular therapy, checkpoint inhibitors, oncolytic viruses and cancer vaccines for the treatment of cancer patients mainly depends on the establishment of effective antigen-specific CD8+ T cells.^[25–27]^ As a result, it is imperative to establish the prognostic model of a CD8+ cell-related signature and evaluate predictability with the mode in HCC.

According to our study, CD8+ T cell infiltration had a significant relationship with the survival of HCC patients. Some studies have reported a relationship between CD8+ T cells and cancer. For example, the accumulation of CD8+ T cells at tumor sites was negatively related to tumor stages and positively related to clinical outcomes in breast cancer.^[28–30]^ The presence of highly increased tumor-infiltrating CD8+ T cells had a favorable prognosis in ovarian cancers, pancreatic, oral squamous cell, and colorectal.^[31–36]^ Next, we obtained 416 CD8Gs that were identified and enriched in immune-related signaling pathways, revealing the potential immune regulation of CD8Gs in HCC.

Next, 5 CD8Gs (KLRB1, FYN, IL2RG, FCER1G, and DGKZ) were established and validated as an independent prognosis risk model. KLRB1 is a favorable prognostic CD8+ T cell signature in pan-cancer.^[37]^ KLRB1 expression in head neck squamous cell carcinoma tissues is markedly correlated with survival. Moreover, KLRB1 levels in peripheral blood mononuclear cells correlate significantly with radiotherapy, and it might be an effective biomarker for assessing nasopharyngeal carcinoma prognosis.^[38]^ FYN has multiple biological functions, mainly in neurological and immune functions.^[39]^ In addition, FYN also serves as a mediator of cell-cell adhesion, integrin-mediated interactions, growth and proliferation, and mitogenic signaling and regulation of cell cycle entry.^[39]^ IL2RG is a common cytokine gamma subunit shared by the receptors of many different interleukins, which display functional redundancy in the development and survival of T and NK cells.^[40,41]^ FCER1G was found to be significantly elevated in malignancy gliomas, and showed a tremendous immunotherapeutic response in glioma patients.^[42]^ FCER1G promotes clear renal cell carcinoma prognosis by influencing immune-related pathways.^[43]^ Multiple myeloma patients with high-expression of FCER1G serve as a prognostic indicator for poor outcomes, which indicates that it might be expected to be implementable as a potential biomarker.^[44]^ DGKZ is considered an oncogene that facilitates the progression and development of cancer.^[45,46]^ DGKZ promotes cancer cell growth and survival by activating the mammalian target of rapamycin complex 1.^[47]^ Although some studies have reported the correlation between these 5 genes and cancers, no studies have reported their prognostic function in HCC. In our study, the HCC patients with high risk also had significantly shorter DFI, DSS, PFI, and OS in stage III/IV, age > 55, age ≤ 55, male, and female, respectively than patients with low risk.

Studies showed that genomic alterations played an important role in various tumors. In this study, the 5-CD8G signature might be related to mutation states in HCC patients. These genomic alterations may drive uncontrolled tumor growth. To meet the metabolic demands of rapid proliferation, the process involves generating aberrant vasculature and consuming oxygen at the early stage of tumor progression.^[48]^ In particular, mutant p53 cooperates with HIF-1 as a promoter of non-small-cell lung cancer progression by hypoxic conditions.^[49]^

TILs play a vital role in disease progression and immunotherapies in many cancers.^[50,51]^ In our study, the 5-CD8G signature was significantly associated with immune cell infiltration and immune-related signal pathways in HCC. A previous study showed that the KLRB1 gene is expressed in most NK cells and a subset of T cells. Here, we found that KLRB1 was positively associated with T cell CD4+ memory resting and T cell CD8+, and not correlated with NK cell activated. The study found that infiltrated monocytes/macrophages induced NK cell activation, and then activated NK cells showed exhaust and death by CD48/2B4 interactions in HCC.^[52,53]^ Furthermore, NK cells interacted with CD8+ T cells which enhanced epitope spreading of the T cell immune response and a tumor antigen-specific T cell immune response in colorectal tumors.^[54]^ Based on KLRB1 expression, the infiltration of CD8+ T cells could not be associated with NK cells to suppress the development of HCC.

The single-cell analysis allows us to identify TILs in the immune microenvironment of HCC. The single-cell analysis was performed to evaluate CD8G expression in HCC. The results showed that 5-CD8Gs, KLRB1, FYN, IL2RG, FCER1G, and DGKZ were more abundantly expressed in the T cell cluster. The status of T cell infiltration and its characteristics are correlated with different prognostic outcomes in cancers.^[55]^ A previous study found that exhaustion markers, such as LAYN, CTLA4, and CXCL13, are associated with T cell exhaustion and prognosis.^[56,57]^ From these results, we hypothesized that the function of CD8Gs in HCC is possibly regulated by activating these subgroups in the HCC microenvironment, which requires further validation.

To improve treatment efficiency, it is important to explore effective drugs to treat patients with HCC. In this study, we investigated whether the CD8Gs risk model could predict chemosensitivity in HCC. The results showed that IC_50_ values are significantly lower in the high-risk subgroup for 10 potentially effective drugs. Although these agents could induce apoptosis in several cancer cells and have vigorous antitumor activity, they were seldom reported to apply in HCC treatment. Undeniably, there are still some limitations in our study that lack validation of laboratory data and clinical data to verify the effects of the 10 anticancer agents on HCC.

## 5. Conclusion

In this study, a 5-CD8G signature was established and validated as a prognostic hallmark of HCC, which was associated with cancer- and immune-related signaling pathways, and the infiltration of immune cells. Drug sensitivity analysis revealed 10 potentially effective drugs for the treatment of HCC patients associated with the 5-CD8G signature. In conclusion, our results might provide important clues for clinical indices to pre-evaluate the efficacy of immunotherapy in HCC patients.

## Author contributions

**Conceptualization:** Xiaozhen Peng, Yiya Zhang.

**Data curation:** Xingjun Lu, Jinyan Liu.

**Formal analysis:** Daqing Yang, Yiya Zhang.

**Methodology:** Honglin Wu, Yiya Zhang.

**Writing – original draft:** Xiaozhen Peng.

**Writing – review & editing:** Hong Peng, Yiya Zhang.

## Supplementary Material

**Figure:**
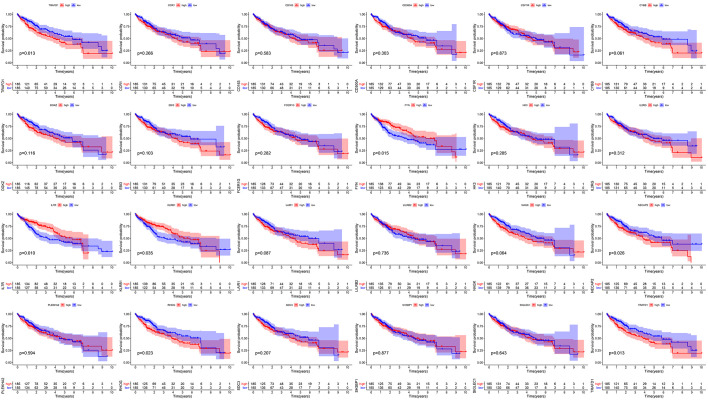


**Figure:**